# Minimally invasive management of hidradenitis suppurativa using a 1470 nm diode laser: a step-by-step description of our technique

**DOI:** 10.1186/s12893-024-02686-8

**Published:** 2025-01-23

**Authors:** Nana Kwame D. Brown, Philemon K. Kumassah, George D. Brown, Solomon Brookmann, Peter C. Ambe, Kwabena Agbedinu

**Affiliations:** 1 Department of Surgery, Korle Bu Teaching Hospital Accra, Accra, Ghana; 2 Department of Surgery, Bank Hospital, Accra, Ghana; 3 Chair of Surgery II, Witten / Herdecke University, Witten, Germany; 4 Department of Surgery, Komfo Anokye Teaching Hospital, Kumasi, Ghana

**Keywords:** Hidradenitis suppurativa, Hidradenitis, Acne inversa, Laser therapy, Diode laser, Dermatology, Laser surgery

## Abstract

**Background:**

The management of hidradenitis suppurativa (HS) requires a multidisciplinary approach to ensure sustainable treatment results, especially in the advanced stages. Traditionally, deroofing and wide excision represented commonly employed surgical techniques. Due to the recurrent nature of HS, tissue preservation should be a relevant aspect of surgical management. The aim of this manuscript is to demonstrate the use of a diode laser for the management of different stages of HS, paying attention to tissue preservation.

**Methods:**

This is a technical manuscript demonstrating our technique for laser–assisted management of HS. A diode laser with a wavelength of 1470 nm was used for this indication. The depth of the sinus/tract dictates the amount of energy required. Our preference is to use 8 Watts for deep lesions and 5 Watts for shallow lesions.

**Results:**

The following 7 critical steps are important to achieve an optimal result with this technique: Drain all collections, minimize tissue damage, protect healthy skin, control risk factors, adopt a multidisciplinary approach, follow up closely, and be patient.

**Conclusion:**

Laser-based management of hidradenitis suppurativa is a promising surgical option in the multidisciplinary treatment of this difficult pathology. The minimally invasive nature of laser surgery, especially tissue preservation, is a strong argument for the role of this technique in the management of this chronic, recurrent condition.

## Introduction

Hidradenitis suppurativa (HS) is a chronic, recurrent, inflammatory condition involving the apocrine glands around the axilla (armpit), breast, groin, perineum, and perianal regions [1]. The condition most commonly affects young individuals from adolescence to early 40s [2]. The disorder is thought to have a multi-factorial genesis with both genetic and epigenetic predispositions. The genetic aspect is backed by its occurrence in specific families with the involvement of many generations, thus supporting a possible autosomal dominant inheritance pattern [3]. Epigenetic aspects of HS constitute well-recognized association with the use of nicotine, obesity and diabetes. Some authors see HS as an autoimmune disorder, like inflammatory bowel disease [4, 5].

The clinical spectrum of presentation is characterized by a chronic, recurrent and eventually progressive behavior, which is best described using the Hurley classification [6]. According to this classification, and its different modifications, grade I disease is characterized by inflammatory, isolated nodules. Left untreated, these nodules may develop into fistulae, some of which get connected (Hurley grade II) [7]. In an attempt to control the recurrent inflammation, scars eventually develop leading to a disfiguring picture (Hurley grade III) [8].

The diagnosis of HS is easily made per visualization [9]. The three primary clinical features that support a diagnosis of Hidradenitis Suppurativa (HS) are:

1. Typical lesions: These include multiple deep-seated inflamed nodules, tombstone comedones, skin tunnels, abscesses, and/or fibrotic scars.
2. Typical locations: The lesions commonly occur in the axillae, groin, and inframammary areas, often presenting in a bilateral distribution.
3. Relapses and chronicity: The condition is characterized by recurring episodes and chronicity [10]. Although histopathology is not routinely needed, it should be considered in selected cases to rule out malignancy, e.g. squamous cell cancer [11]. Inspection of all predilection sites should be done in all patients suspected of or presenting with HS [12].

The management of HS should be multidisciplinary including both conservative or medical strategies and surgery [13, 14]. Many international guidelines suggest a stepwise, grade-guided treatment algorithm including topical and systematic antibiotics, hormone-directed strategies, immune modulation, and surgery [14].

The surgical strategy may range from a limited procedure like deroofing to extensive procedures like wide excision and fecal deviation with or without plastic reconstruction [15]. The laser techniques constitute an item within the surgical armamentarium [16]. The aim of this manuscript is to describe our technique of minimally invasive management of HS using a diode laser and to discuss this technique with respect to the available literature.

## Methods

This is a technical manuscript demonstrating our technique for laser–assisted management of HS.

We use a diode laser (Biolitec, Germany) with a wavelength of 1470 nm in the continuous mode for this indication. The depth of the sinus/tract dictates the amount of energy required. The laser energy is chosen depending on the depth of the lesion or tract, which can be estimated using the scale on the laser probe or indirectly via the brightness of the indicator light at the tip of the laser probe. For optimal treatment results, a laser setting should be chosen to enable sufficient energy application within the lesion or tract without causing thermal damage to the overlying skin. Our preference is to use 8 Watts for deep lesions and 5 Watts for shallow lesions, and the laser is fired in a retrograde fashion. The laser energy is emitted at the tip of the probe in a circular fashion leading to denaturation of the proteins along the inflammatory tracts. This combined with gentle compression causes the tract to collapse and close. The laser penetrates the skin to selectively target and thermally destroy the follicular unit and organized inflammatory lesions within the superficial to mid-dermis [17]. The procedure can be performed with general or local anesthesia as needed. The pathologic lesion is punctured and gently curetted. Attention must be paid to the size of the debriding instrument to prevent dilating the tract. Irrigation can be done in selected cases.

## Results

**Fig. 1:**
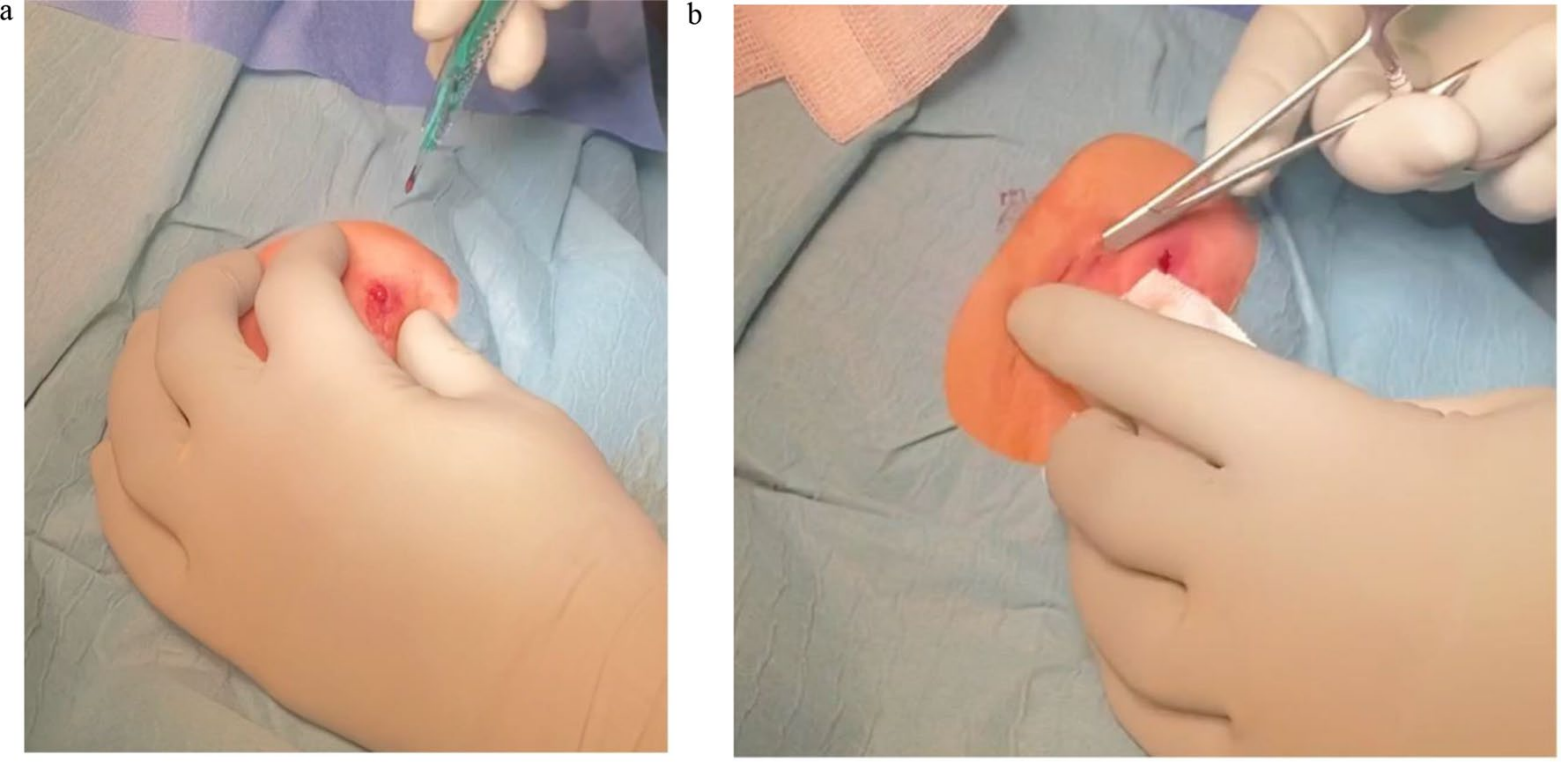
(**a**) Incision and Release of Collection (left axilla). (**b**) Gentle Debridement with a Mosquito Clamp (left axilla)

**Fig. 2:**
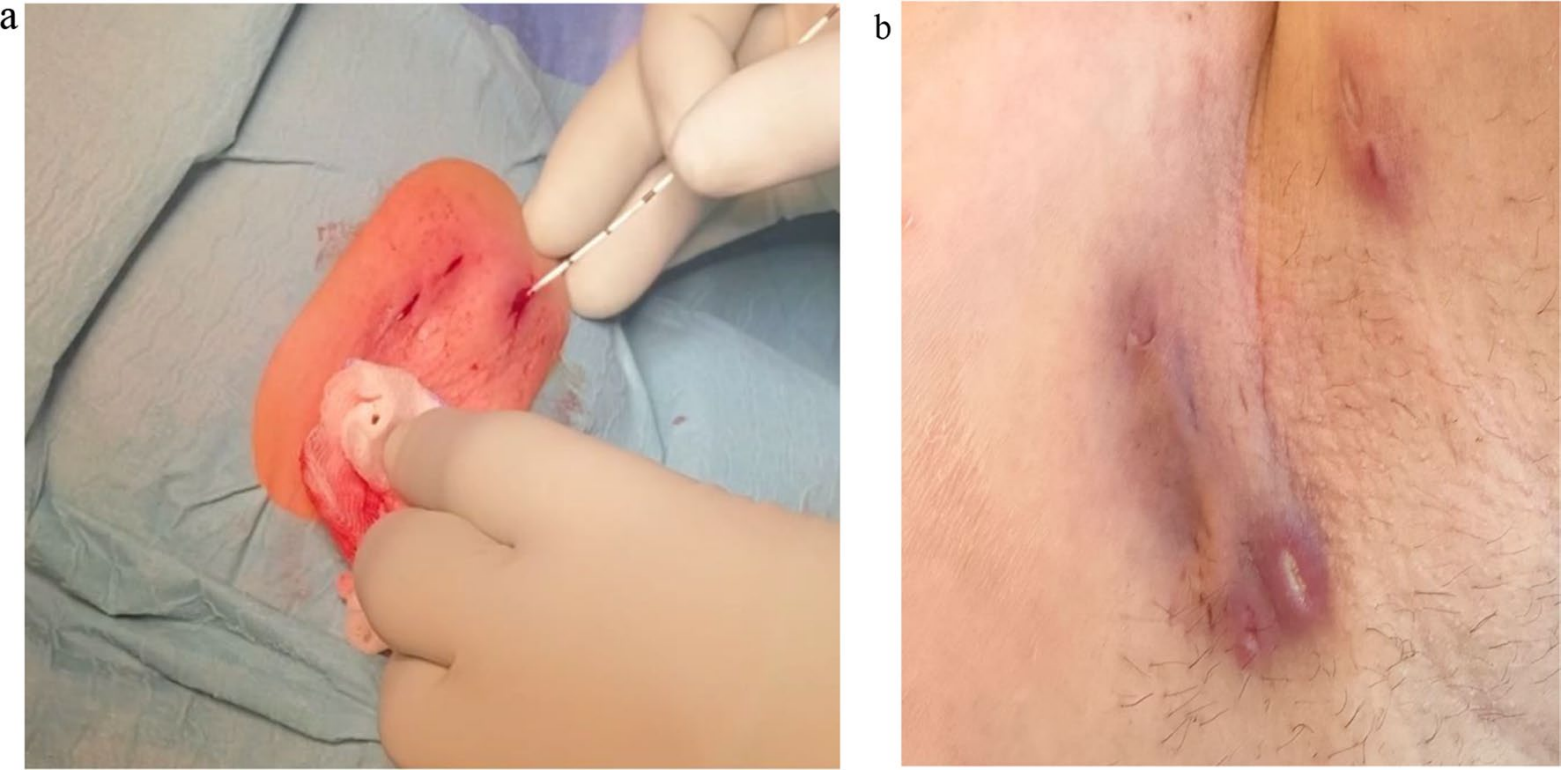
(**a**) Applying Cold Gauze to Healthy Skin (left axilla). (**b**) Day 7 Post-Op (left axilla)

The following seven points should be considered when managing these patients:

1. **Draining all collections**: This is best achieved via a small incision at the apex of the collection prior to laser application. In cases with large collections and purulent secretion, laser surgery should be omitted. A gentle debridement of the tract may be performed. Irrigation is optional (Fig. 1a and b).

2. **Minimalize Tissue Damage**: This is achieved by making small incisions for drainage and by regulating the amount of laser energy applied to the tissue.
3. **Protect Healthy Skin**: Superficial tracts within the subcutis may be covered just by a thin skin layer. This healthy skin should be protected from thermal damage via reducing the amount of laser energy applied and by applying a wet/cold gauze on the area during treatment (Fig. 2a).

4. **Control Risk Factors**: Smoking is a relevant risk factor for HS and quitting smoking may significantly influence the course of HS and treatment success.
5. **Multimodal Approach**: This includes but is not limited to the use of prolonged antibiotics, antihormonal therapy, and biologics.
6. **Close Follow-Up**: The chronic and recurrent nature of HS requires a close follow-up. (Fig. 2b: Day 7 Post-Op)
7. **Patience**: Healing post-op may take some time.

The surgery can be performed both under general or local anesthesia; even as an outpatient procedure. Postoperative wound dressing is done with a simple gauze. Postoperative pain is minimal and return to work is possible on Post-Op Day 1.

**Fig. 3:**
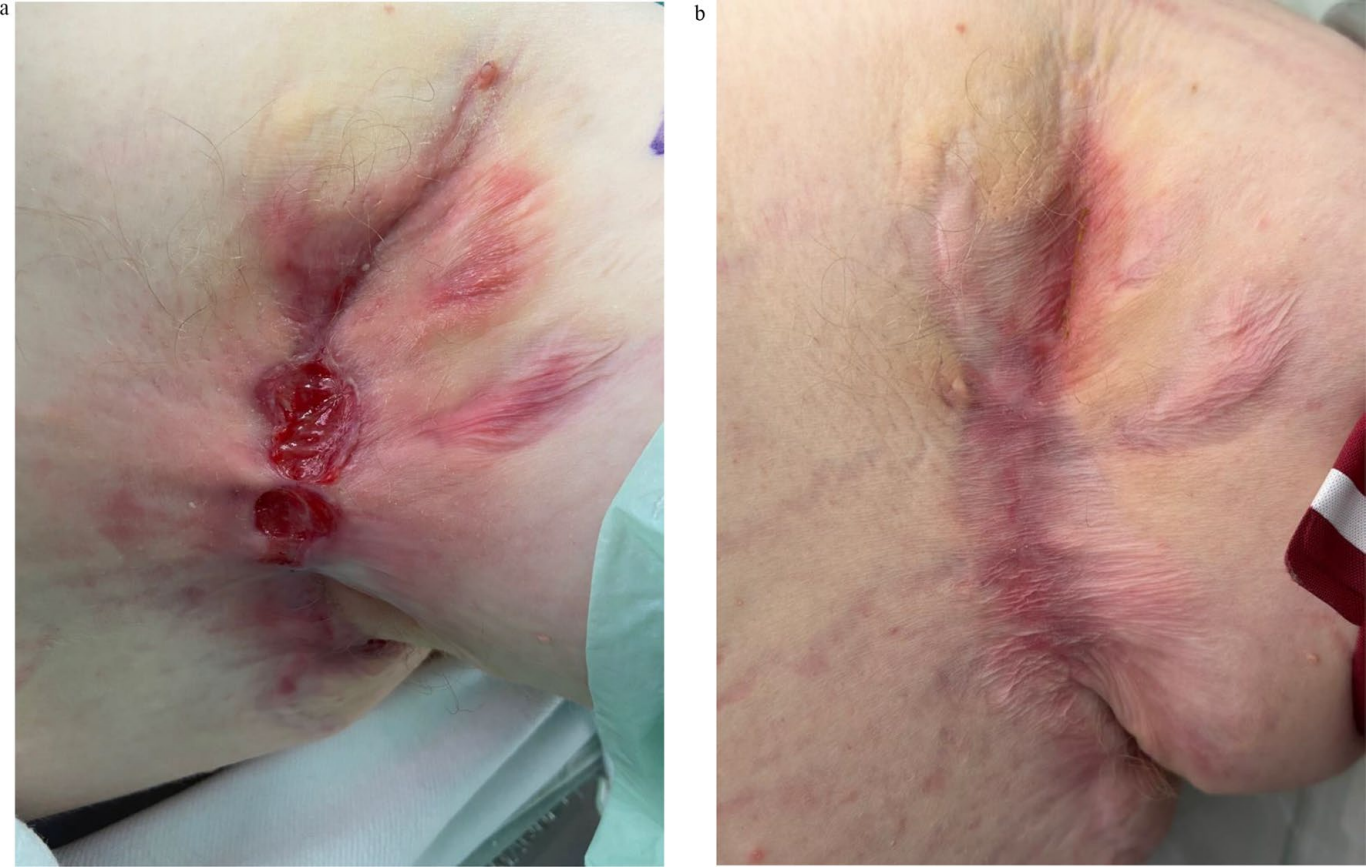
(**a**) Extensive Disease - Before Laser Treatment (left axilla). (**b**) Extensive Disease - After 2 Laser Sessions (left axilla)

**Fig. 4:**
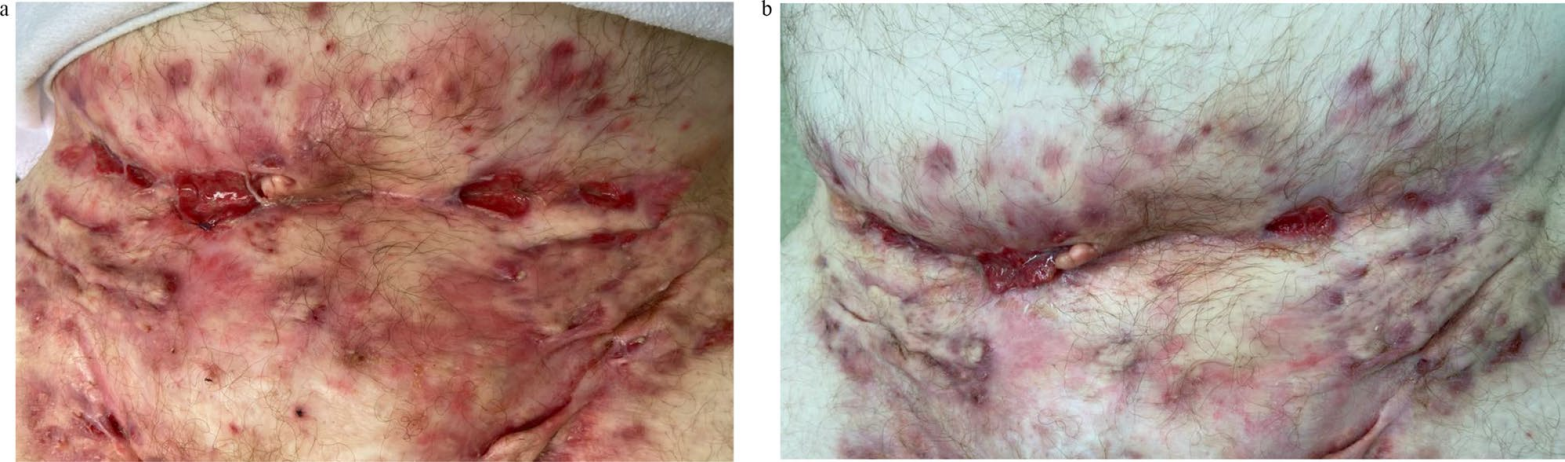
(**a**) Extensive Disease (2) - Before Laser Treatment (low abdomen / inguinal). (**b**) Extensive Disease (2) - After 2 Laser Sessions (lower abdomen / inguinal)

Extensive disease usually requires more than one treatment session and additional treatment such as the use of biologics (Figs. 3 and 4).

## Discussion

Over many decades, the surgical management of HS ranged from limited local procedures like incision and drainage, and deroofing to more extensive resections, with additional procedures like creating a stoma for fecal deviation. Some of these procedures led to excellent control at high costs for the patients with regard to the risk of morbidity and quality of life. Thus, the need for less invasive surgical options with similar disease control became apparent.

This manuscript presents our technique for performing laser surgery for patients with HS. This technique uses a well-defined amount of laser energy to destroy the epithelial lining of sinuses and tracts seen in patients with HS. This results in the denaturation of proteins in the sinuses and tracts, which subsequently collapse and close secondary to the adhesive effect of the denaturized proteins.

The use of laser in the management of HS is a well-recognized approach that has been stated in numerous treatment recommendations and guidelines. In the 2016 European guidelines for HS, Laser treatment was recommended for Hurley II and III [18]. Laser treatment in these guidelines reached a strong recommendation due to the high evidence level from the randomized controlled trial by Tierney et al. using the Nd: YAG laser [16].

In recent years, more appealing data has been reported for the use of laser technology in HS. While some of this data is related to the effect of hair removal in patients with HS, there is nonetheless increasing experience with the use of this modality to manage this rather challenging pathology [19–21].

Our experience with laser-associated treatment of patients with HS is similar to findings from the current literature. However, the multifaceted nature of Hydradenitis warrants a multidisciplinary approach. Therefore, we routinely use a combination of treatment options in the management of our patients. Our standard algorithm is to combine laser with a long course of antibiotics (clindamycin 600 mg and rifampicin 600 mg daily for 12 weeks) in patients with Hurley stages I and II and to escalate to a biologic in patients who are unresponsive to and do not tolerate antibiotics [14]. Also, we recommend biologics in patients with extensive disease and those with Hurley stage III [14]. We have observed so far that almost all our patients with stage I would need just a single laser intervention for good control. Patients with stages II and III may require repeated intervention. In such cases, we recommend repeated surgeries at 6–8 week intervals.

While most data in the literature report on the use of either the CO_2_ or the Nd: YAG laser, our experience is based on the diode laser. Thus, a direct comparison may be questionable.

Irrespective of the above limitation, the minimally invasive laser surgery for HS largely preserves the tissue. Laser surgery has been shown to significantly reduce postoperative pain and morbidity, leading to an early return to work and a better quality of life. It would be interesting to study the positive experiences reported in this manuscript in a prospective setting with more patients.

## Conclusion

Laser-based management of hidradenitis suppurativa is a promising surgical option in the multidisciplinary treatment of this difficult pathology. The minimally invasive nature of laser surgery, especially tissue preservation, is a strong argument for the role of this technique in the management of this chronic, recurrent condition.

## Data Availability

All data has been included in the manuscript. Further questions should be forwarded to the corresponding author.

## Abbreviations

HS: Hidradenitis suppurativa
